# Ultrasensitive, rapid and inexpensive detection of DNA using paper based lateral flow assay

**DOI:** 10.1038/srep37732

**Published:** 2016-11-25

**Authors:** Miriam Jauset-Rubio, Markéta Svobodová, Teresa Mairal, Calum McNeil, Neil Keegan, Ayman Saeed, Mohammad Nooredeen Abbas, Mohammad S. El-Shahawi, Abdulaziz S. Bashammakh, Abdulrahman O. Alyoubi, Ciara K. O´Sullivan

**Affiliations:** 1 Nanobiotechnology and Bioanalysis group, Department of Chemical Engineering, Universitat Rovira I Virgili, 43007 Tarragona, Spain; 2 Institute of Cellular Medicine, Diagnostic and Therapeutic Technologies Group, Newcastle University, Newcastle upon Tyne, NE2 4HH, UK; 3 National Research Centre, Cairo, Egypt; 4 Department of Chemistry, Faculty of Science, King Abdulaziz University, P. O. Box 80203, Jeddah 21589, Kingdom of Saudi Arabia; 5 Institució Catalana de Recerca I Estudis Avancats, Passeig Lluís Companys 23, 08010 Barcelona, Spain

## Abstract

Sensitive, specific, rapid, inexpensive and easy-to-use nucleic acid tests for use at the point-of-need are critical for the emerging field of personalised medicine for which companion diagnostics are essential, as well as for application in low resource settings. Here we report on the development of a point-of-care nucleic acid lateral flow test for the direct detection of isothermally amplified DNA. The recombinase polymerase amplification method is modified slightly to use tailed primers, resulting in an amplicon with a duplex flanked by two single stranded DNA tails. This tailed amplicon facilitates detection via hybridisation to a surface immobilised oligonucleotide capture probe and a gold nanoparticle labelled reporter probe. A detection limit of 1 × 10^−11^ M (190 amol), equivalent to 8.67 × 10^5^ copies of DNA was achieved, with the entire assay, both amplification and detection, being completed in less than 15 minutes at a constant temperature of 37 °C. The use of the tailed primers obviates the need for hapten labelling and consequent use of capture and reporter antibodies, whilst also avoiding the need for any post-amplification processing for the generation of single stranded DNA, thus presenting an assay that can facilely find application at the point of need.

Lateral flow assays (LFA) are very simple and highly successful rapid analytical platforms derived from the latex agglutination test developed by Singer and Plotz in 1956 for the serological diagnosis of rheumatoid arthritis^1^. Lateral flow, or immunochromatographic, tests were first reported in the early 1980 s^2^ and were commercially launched by Unipath in 1984 with the first product being a urine-based pregnancy test^3^. Since then, hundreds of lateral flow assays have been reported and commercialised with applications for detection of infectious diseases, cancer, cardiac diseases, toxins, pathogens, pesticides and metal ions as well as for pharmaceuticals and drugs, as has been reviewed extensively^4^,^5^.

LFAs are typically composed of a nitrocellulose membrane, sample pad, conjugate pad, wicking or absorbent pad and backing pad^6^. Nitrocellulose membranes are most commonly used as they facilitate a support capable of use for both reaction and detection, with capture biomolecules e.g. antibodies, are deposited on the nitrocellulose to form the test and control lines via a combination of electrostatic interactions, hydrogen bonds and/or hydrophobic interactions^7^. The point-of-care (POC) market is rapidly expanding, believed to be worth US$15 billion in 2011 and predicted to have an annual compound growth of 4% to reach US$18 billion by 2016^8^. The World Health Organisation has provided guidelines for these POCTs, which are referred to as ASSURED (Table 1).

Nucleic acid testing has important applications in food safety analysis, environmental monitoring and increasingly in medical diagnostics. Meeting the emerging paradigm of medicine, in which pharmacogenomics and individualised theranostics are of increasing importance for patient stratification and avoidance of adverse drug effects, there is a clear need for rapid, inexpensive, highly sensitive and simple-to-use companion diagnostic tests for the qualitative/quantitative detection of nucleic acids^9^. Meeting this requirement, there are a large number of paper analytical devices (PAD) that have been developed for detection of PCR products using lateral flow assays. There are two mains types of lateral flow nucleic acid tests, referred to as Nucleic Acid Lateral Flow (NALF) and Nucleic Acid Lateral Flow ImmunoAssay (NALFIA); NALF directly detects DNA exploiting capture and labelled reporter oligonucleotide probes, whereas NALFIA detects hapten-labelled DNA using capture and labelled reporter antibodies or streptavidin. The first example was a NALFIA, reported in 2000 for the detection of *Cryptosporidium parvum*, in which the authors described the use of biotin and fluorescein isothiocyanate (FITC) labelled forward and reverse primers for PCR and detected the duplex using immobilized anti-FITC antibodies and streptavidin coupled to coloured latex microparticles^10^. This was quickly followed by a similar approach for the detection of *Staphylococcus aureus -* in this case using immobilized streptavidin and anti-fluorescein antibodies labelled with gold nanoparticles^11^. Since then numerous NALFIAs have been reported using different hapten labels, including digoxigenin (dig), carboxyfluorescein (FAM), FITC and biotin^4^. There are far fewer examples of NALF, which can be attributed to the kinetics of hybridisation in lateral flow being far more complex compared with the formation of hapten-antibody complexes. Corstjens *et al*. first reported the use of up-converting phosphor technology (UPT) reporters using a signal enhancement tool in a NALFIA using dig and biotin hapten labels and subsequently modified the approach for the detection of an asymmetric PCR product using a biotinylated capture probe immobilised via streptavidin coated on the nitrocellulose strip and a UPT labelled reporter probe, representing the first example of a NALF^12^. In the same year, Glynou *et al*.^13^ reported the first example of a NALF, in which a biotinylated oligonucleotide probe was used as a capture probe, and an oligonucleotide probe labelled with gold nanoparticles was used as a reporter, but required enzymatic tailing of probes. Soon after, Baeumner *et al*.^14^ detailed the use of a liposome labelled oligonucleotide reporter probe and biotinylated capture probes, using polyethersulphone membranes, with the liposome-oligo complex being formed off strip and subsequently wicked to the detection zone. Liu’s team^15^,^16^ has published a series of papers detailing various formats of NALF, achieving a LOD of 0.5 nM using gold nanoparticles and improving this LOD via the use of horseradish peroxidase adsorbed on gold nanoparticles linked to the reporter probe as a means of signal amplification, in both cases for a synthetic DNA target. They subsequently reported the simultaneous lateral flow detection of proteins and nucleic acids, again using oligo capture and reporter probes^17^ and recently improved on these previous reports, using carbon nanotube labelled reporter probes, and using pre-mixed streptavidin-biotinylated probe as the capture probe, achieving an LOD of 40 pM for a synthetic DNA target^18^.

Whilst PCR is the most commonly reported method of amplification combined with lateral flow^19^,^20^,^21^, there are an increasing number of reports combining isothermal amplification with lateral flow detection^22^,^23^,^24^,^25^,^26^,^27^,^28^,^29^,^30^, moving nearer to achieving ASSURED devices that can truly be used at the point-of-need.

Isothermal amplification offers the possibility of being able to carry out on-site analysis of DNA, and several techniques have been reported and exploited in the last decade either carrying out amplification followed by detection, or combining amplification and detection such as the nucleic acid sequence based amplification (NASBA), transcription mediated amplification (TMA), self-sustained sequence replication (3SR), helicase dependent amplification (HDA), rolling circle amplification (RCA) and loop mediated isothermal amplification (LAMP)^31^,^32^,^33^,^34^,^35^,^36^,^37^,^38^,^39^. Devices using LAMP which integrate purification, amplification and detection have been reported^40^, but these devices use fluorescence or turbidity measurements, which limits the application of LAMP for multiplexing. Furthermore, LAMP is highly dependent on the extremely careful design of multiple complex primers^41^. The recombinase polymerase amplification (RPA) is a very attractive alternative that overcomes all of the drawbacks of the other isothermal approaches^42^. An increasing number of reports detailing different formats of RPA are appearing and the technique gives true promise for application at the point-of-need via sensors or lateral flow formats^43^,^44^,^45^,^46^,^47^,^48^,^49^,^50^,^51^,^52^,^53^,^54^,^55^.

There are a plethora of reports detailing the combination of isothermal amplification with lateral flow detection - the vast majority exploiting LAMP and almost exclusively NALFIA formats, with some exceptions, such as the detection of the single stranded DNA products from a two-stage exponential amplification reaction (EXPAR) using a biotinylated capture probe immobilized on Neutravidin and a microsphere bead labelled reporter probe^23^. The use of LAMP combined with NALF has also been reported, but requires a post-LAMP denaturation step at 95 °C prior to detection^9^.

The goal of this study was to develop a point-of-care nucleic acid lateral flow test for the direct detection of RPA products. Tailed primers are exploited resulting in an amplicon with a duplex flanked by two single stranded DNA tails - allowing detection via hybridisation to a surface immobilised oligonucleotide capture probe and a gold nanoparticle labelled reporter probe. Assay parameters were optimised and a range of target DNA concentrations tested. The use of the tailed primers obviates the need for hapten labelling and consequent use of capture and reporter antibodies, whilst also avoiding the need for any post-amplification processing for the generation of single stranded DNA, thus presenting a generic assay platform that can find application at the point-of-need.

## Results and Discussion

**Figure 1:**
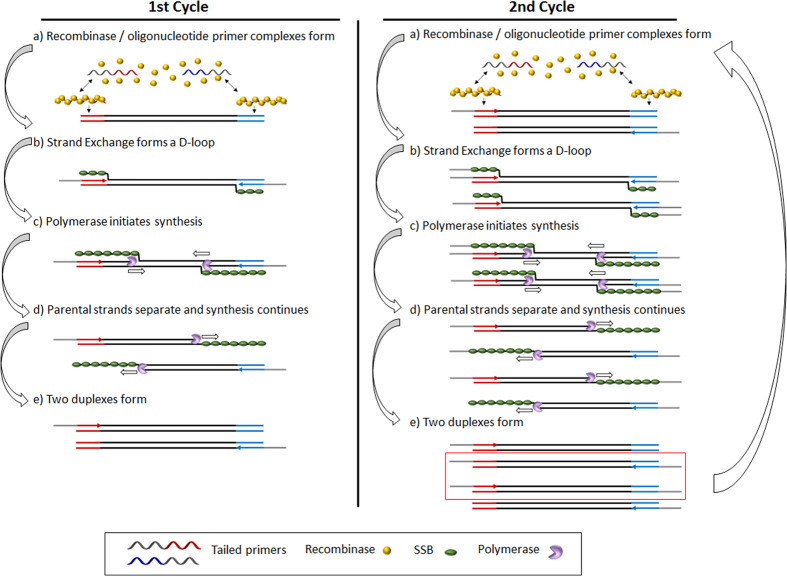
Schematic representation of liquid-phase RPA with tailed primers.

In RPA, the need for thermal cycling used in the polymerase chain reaction is avoided and replaced by three core proteins that operate optimally between 37 °C and 40 °C. The first protein, a recombinase, binds to primers, forming filaments that can then recombine with homologous DNA in a duplex target, forcing displacement of the non-complementary strand and thus provoking the formation of a D-loop. The second protein is a single-stranded DNA binding protein, which attaches to the strand of DNA displaced by the primer, preventing the dissociation of the primer and hybridisation of the duplex target. The final core protein is a strand-displacing polymerase that copies the DNA, adding bases onto the 3′ end of the primer, forcing open the double helix as it progresses. When opposing primers are used, exponential amplification occurs^42^ (Fig. 1).

The combination of RPA and lateral flow detection has been reported since 2013, and the use of normal physiological temperature to perform amplification has been demonstrated^43^. In addition, RPA-LF has been used for the detection of HIV-1^44^,^45^, Canine Visceral Leishmaniasis^46^, *Orientia tsutsugamushi*, *Rickettsia typhi*^47^, plasmodium^48^, intestinal protozoa^49^, *cryptosporidiosis*^50^, Yellow Fever Virus^50^, *Penaeus stylirostris*^24^, *Plasmodium falciparum*^26^, *Entamoeba histolytica*^51^, *Schistoma haematobium*^29^, little cherry virus^52^ and plum pox virus^53^. All these reports exploit RPA amplification followed by lateral flow strip detection based on the use of two different kits. The first is the TwistAmp nfo kit (TwistDX) combined with commercial Hybridetect strips (Milena)^24^,^26^,^29^,^44^,^45^,^46^,^47^,^48^,^49^,^50^,^51^,^54^,^55^ and the other is the Amplify RP kit (Agdia)^52^,^53^. In both cases antibodies are used as capture probes in test and control lines and a probe modified with a hapten, sometimes a fluorophore such as FAM, was used as a reporter probe. The lateral flow strips are read with a commercial scanner in combination with specific software to achieve better detection limits.

Taking in account previous reported work in our group based on the use of RPA compared with PCR^56^, it was performed a rapid time-dependent assay to check if adding the tail primers, the optimal conditions achieved before would change. Different assay times (5, 10, 15, 20, 25, 30, 35, 40, 45 minutes) were monitored and the samples were run in agarose gel to compare the intensities of the bands, demonstrating that 15 minutes is enough time to get amplification as not difference it was observed with higher times of assay (Supplementary Fig. S1).

**Figure 2:**
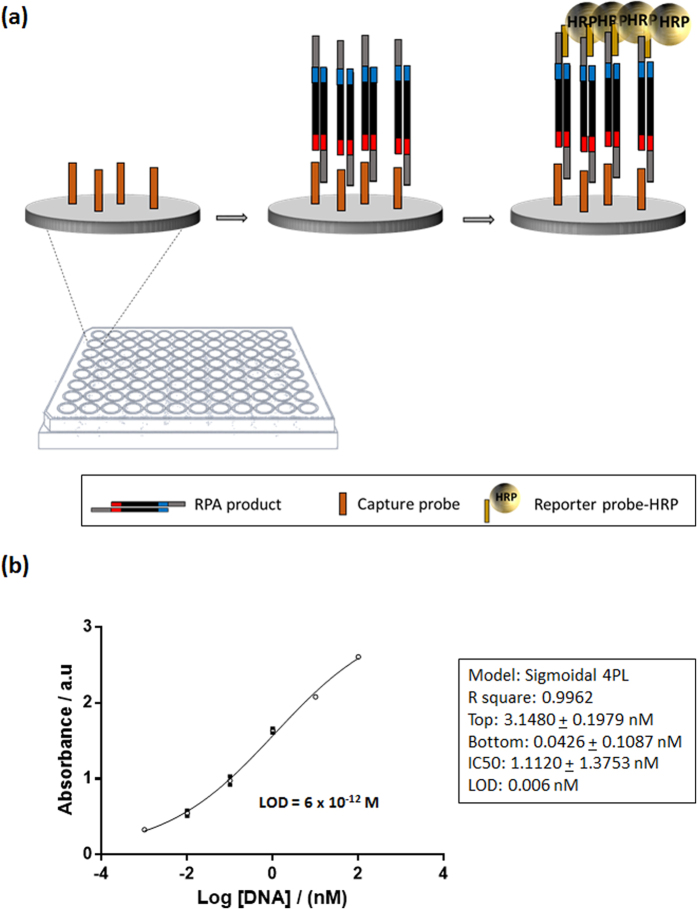
Maleimide coated microtiter plate assay. (**a**) Schematic representation of hybridisation of immobilised capture probe and HRP reporter probe with single stranded tailed RPA amplicon; (**b**) Calibration curve using different amounts of RPA DNA amplified using tailed primers.

As a proof of concept for the detection of DNA amplified via RPA using tailed primers, maleimide coated microtiter plate was used to immobilise thiolated capture probe. RPA amplicons, duplex flanked by two single stranded tails, were added to the wells of the microtiter plate and hybridised to the immobilised capture probe, and then to the reporter probe conjugated with horseradish peroxidase (HRP) (Fig. 2a). The tailed amplicon was successfully detected and the LOD calculated by GraphPad Prism software, defined as the blank plus three times the standard deviation of the blank, was calculated to be 6 × 10^−12^ M (Fig. 2b).

Different methods have been evaluated for the preparation of the reporter probe conjugated with gold nanoparticles^57^,^58^,^59^. Co-immobilization was explored as a means of controlling the probe surface density, as it is well known that spacing between the immobilised probes minimises steric hindrance, favouring accessibility to the complementary DNA strand. The ratio usually used for DNA to the short alkane thiol, mercaptohexanol (MCH) ranges between 1:10 and 1:100^60^. Additionally, to ensure that the thiol moiety on DNA is free, treatment with reducing agents such as DTT or TCEP is often incorporated in the conjugation protocol, thus breaking any disulphide bridges that may have formed between probes. Some reports claim that the best results are achieved with DTT, meanwhile others prefer TCEP due to the fact that it does not compete or react with thiolated compounds, thereby eliminating the need to remove it prior to conjugation with AuNPs^61^. Finally, the direct immobilization of the DNA probe on to AuNPs, with no co-immobilized alkane thiol or use of reducing agents, was tested. Thiolated DNA 1 and DNA 2 (DNA 1 incorporating a 15-mer poly-T spacer) were compared.

**Figure 3:**
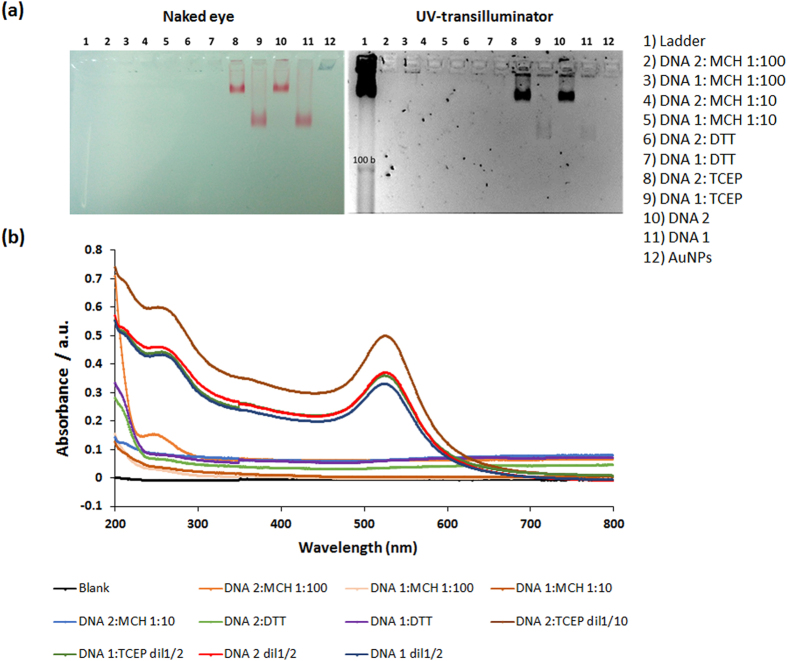
Analysis of reporter probe-AuNP conjugation using. (**a**) Agarose gel image by naked eye and by UV-transilluminator, highlighting unsuccessful DNA-AuNP conjugation using MCH co-immobiliser (Lanes 2–5) or DTT as reducing agent (Lanes 6–7), whilst successful conjugation is demonstrated via the slower gel migration of DNA-AuNP conjugates without immobiliser/reducing agent (Lanes 10, 11), with the highest level of AuNP loading with DNA observed using TCEP as reducing agent (Lanes 8, 9); (**b**) Spectrophotometer analysis of gold nanoparticle-DNA conjugates highlighting peaks obtained at 260 nm (DNA) and 520 nm (AuNP).

To characterize the different methodologies two strategies were studied. 1× TBE agarose gel 3% was prepared to carry out electrophoresis analysis of the prepared conjugates, allowing analysis and by naked eye as well as by UV following staining with GelRed. As can be seen in Fig. 3a. in Lanes 2–5, the use of mercaptohexanol as a co-immobiliser, without pre-treatment with any reducing agent, did not result in successful DNA-AuNP conjugation. Likewise, the use of a DTT reducing agent was not observed to result in successful conjugation, perhaps due to the DTT competing with the thiolated DNA for immobilization. However, a visual difference was observed in the case of TCEP pre-treatment to reduce any disulphide bonds, as well as direct conjugation with both DNA probes, with better results in both cases being observed for DNA 2, the DNA reporter with an incorporated 15 T spacer. Free DNA unconjugated to the AuNPs would be expected to migrate to lower than the 100 bases band of the ladder, but as can be seen for both the DNA 1 and DNA 2 reporter probes, there is a clear retardation in their migration along the gel. This can be attributed to both the increased size of the DNA-AuNP complex, as well as to each AuNP bearing multiple copies of the DNA probe. Furthermore, it can be observed that the AuNPs linked to the DNA 2 probe bear far more DNA than the AuNPs linked to DNA 1, presumably due to the 15 T spacer allowing an optimal spatial orientation of the DNA 2 probe on the AuNP surface, thus facilitating increased surface accessibility for enhanced chemisorption for a higher number of DNA probes.

The DNA-AuNPs conjugates were also evaluated using UV-visible spectroscopy, by scanning the wavelength from 200 to 800 nm. Two peaks, one at 260 nm for DNA and another at 520 nm for AuNPs, are expected to be observed. TCEP pre-treatment and direct immobilization in the absence of any co-immobilizer were again observed to provide the optimal conjugates (Fig. 3b). Again, pre-treatment with DTT resulted in no linkage of DNA to the AuNP, but this could be attributable to the DTT competing with the thiolated DNA for chemisorbing to the gold.

### Lateral flow assay

**Figure 4:**
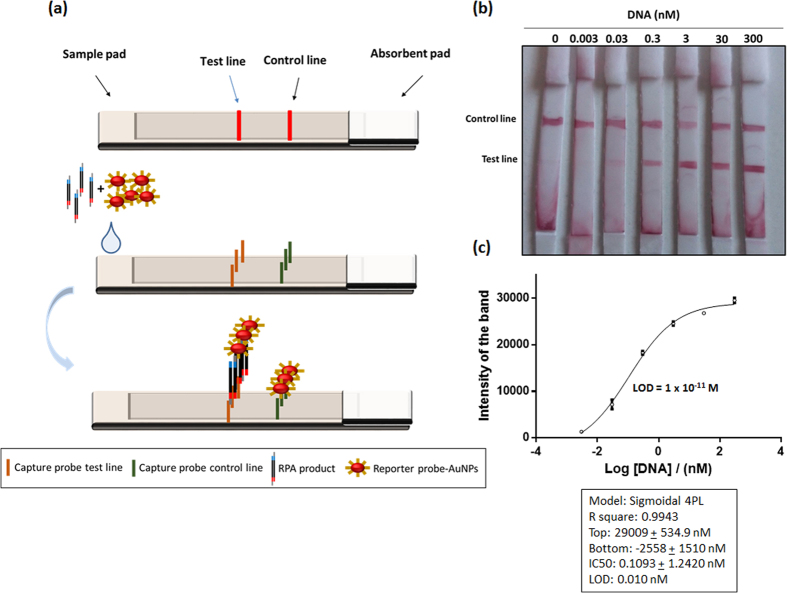
Lateral flow assay. (**a**) Schematic representation of RPA-NALF; (**b**) Images of NALFs with varying concentrations of RPA amplified DNA; (**c**) Extrapolated calibration curve and LOD of the assay.

Once detection of the duplex flanked by single stranded tails via hybridisation to a surface tethered capture probe and a labelled reporter probe had been demonstrated using a microtiter plate format, the system was successfully transferred to a lateral flow assay format. The lateral flow was based on the immobilization of two biotinylated capture probes for each of the test and control lines. On the test line, the immobilised probe is complementary to the tail in the 5′ region of the amplified DNA, meanwhile in the control line, the probe is complementary to the reporter probe conjugated with AuNPs. This reporter probe is also complementary to the other tail on the 3′ end of the amplified, tailed DNA. Thus, the reporter probe conjugated with gold nanoparticles bound to the amplified DNA forming a sandwich on the test line and with the capture probe on the control line, generating in both cases a red line visible to the naked eye (Fig. 4a).

In order to improve assay performance, three different membranes were evaluated to find the optimal material for creation of the lateral flow strips. Unistart CN95 is based on cellulose nitrate, and has large pores, with a rapid flow rate from 90–135 s/4 cm. This membrane is recommended for blood or serum tests being applicable to the detection of cells and bacteria. Alternatively, FF170HP is a nitrocellulose membrane ideal for use with low viscosity samples with a flow rate of 156 s/4 cm. Finally, Biodyne B membrane, a nylon membrane which contains exclusively positive charges, providing the highest possible binding capacity for negatively charged molecules such as nucleic acids. This membrane was used to immobilize the capture probes directly onto the membrane without DNA modification, but no successful results were observed, which may be attributed to the DNA being linked planarly to the membrane surface, rendering it inaccessible for hybridisation with the tailed target. The best option was FF170HP membrane, which resulted in control and test lines of the highest intensities.

Different approaches for immobilisation of the streptavidin and the biotinylated capture probes on the membrane were evaluated. Three methodologies were compared: (a) dispensing streptavidin, allowing to dry, followed by addition of the biotinylated capture probe; (b) pre-incubation of streptavidin with the biotinylated capture probe, and dispensing of the pre-formed complex onto the membrane; (c) pre-incubation of streptavidin with the biotinylated capture probe followed by centrifugation using 30 kDa cut-off Microcon to eliminate the excess biotinylated capture probe before dispensing of the pre-formed complex onto the membrane. The most intense bands where observed with pre-incubation without centrifugation.

In order to establish the sensitivity of the NALF, different concentrations of DNA were amplified using RPA and detected, with the test line being visible to the naked eye at concentrations as low as 30 pM (Fig. 4b). Built-in cameras in mobile phones have been used as imaging platforms^62^,^63^ as well as for the detection of disease biomarkers and infectious pathogens^64^,^65^,^66^,^67^,^68^,^69^, and in this work a Smartphone camera was used to take an image of the strip followed by an application based on Image J software termed IJ_mobile to calculate the intensity of the bands, and these values were plotted using GraphPad Prism software in order to obtain the LOD of the assay. The data was normalized by subtracting the value obtained in the blank, and the LOD achieved was 1 × 10^−11^ M, with a complete assay, combining amplification and detection at 37 °C, taking 15 minutes to complete.

The combination of the tailed primers with isothermal recombinase amplification is a very elegant solution for rapid, cost-effective and highly sensitive nucleic acid lateral flow assays. The entire assay, including amplification and detection, was completed in just 15 minutes, which is a considerable improvement on the majority of the reported combinations of amplification and detection, which typically require at least 45 minutes. This is attributable both to the efficiency of the RPA, as well as the rapid hybridisation kinetics of the tailed amplicon with the surface immobilised probe and the reporter probe. DNA hybridisation kinetics are based on a first step of collisional kinetics, in which the DNA target randomly collides with the DNA probe and a discrete state where complementarity between some bases are found – this complementarity may rapidly dissociate and the collisional kinetics proceed until better complementarity is found between target and probe, followed by a second step of a rapid DNA zippering process. The single stranded tails render enhanced kinetics as there is less requirement for collisional kinetics due to a lower number of bases being available for hybridisation. In practice, it was observed visually that hybridisation was virtually instantaneous - with the red colour forming at the test line in just 30 seconds following sample addition. The additional time was simply to allow all non-incorporated reporter probes to wick to the absorbent pad. Furthermore, the tailed primers facilitated the use of oligonucleotide capture and reporter probes, avoiding hapten labelling and antibodies - thus having a significant impact on cost. This could also be expected to have a marked impact on storage stability time as well as storage requirements. The costs/strip, on a laboratory research scale for the RPA-NALF reported here, is 1.15€, compared with 5.22€ for hapten-antibody NALFIA, 4.14€ for hapten-protein (i.e. streptavidin), or 4.37€ per strip for the Milenia Hybridetect kit (Table 2).

Future work will focus on directly functionalising the nitrocellulose membrane with the DNA capture probe, as well as integrating amplification and detection on a single lateral flow assay. Real-time and accelerated storage stability studies will also be carried out.

## Conclusions

We have reported the first example of a recombinase polymerase amplification-nucleic acid lateral flow, exploiting tailed primers which result in duplex amplicons flanked by single stranded DNA tails. These DNA tails facilitate extremely rapid hybridisation with an AuNP labelled reporter probe, as well as an immobilised capture probe, and contribute not only to a decreased assay time, but also to a markedly reduced assay cost. The combination of RPA, tailed primers and a nucleic acid lateral flow system address the requirement for ASSURED diagnostics at the point-of-need.

## Materials and Methods

### Materials

Phosphate buffered saline (PBS, 10 mM phosphate, 138 mM NaCl, 2.7 mM KCl, pH 7.4), PBS-Tween (10 mM phosphate, 138 mM NaCl, 2.7 mM KCl, 0.05% v/v Tween 20, pH 7.4), 1-ethyl-3-(dimethylaminopropyl) carboiimide (EDC), N-hydroxysuccinimide (NHS), and all other reagents were purchased from Sigma (Barcelona, Spain). Magnesium chloride, sodium chloride, sodium hydroxide and hydrochloric acid were purchased from Scharlau Chemie S.A. (Barcelona, Spain). Pierce^TM^ maleimide activated plates, 8-well strip, were from Pierce (Madrid, Spain) and DNA oligonucleotides were purchased from BIOMERS (Ulm, Germany). All primers and probe sequences can be found in Table 3.

### Preparation of microtiter plates

Maleimide plates were prepared by pipetting 100 μl of 200 nM thiolated capture probe prepared in PBS and left to incubate overnight at 4 °C. The plates were subsequently washed with PBS-Tween and any remaining maleimide groups were blocked with 100 μM 6-mercapto-1-hexanol in deionized water adding 200 μl per well for 1 hour before washing the plate thoroughly with PBS-Tween.

### Recombinase Polymerase Amplification (RPA) reaction

RPA was performed in a tube following the indications provided in the TwistAmp Basic kit (TwistDX, Cambridge, UK). Briefly, master mix was prepared in a tube with 480 nM of each primer, template duplex DNA (94 base pairs) of the desired concentration, 14 mM magnesium acetate and 1× rehydration buffer. The reaction proceeded at room temperature for 20 minutes/37 °C for 15 minutes.

### Enzyme Linked Oligonucleotide Assay

The resulting RPA product was added to the functionalised maleimide plates (50 μl per well) for 30 minutes at room temperature under shaking conditions, followed by a washing step with PBS-Tween, and subsequent addition of 50 μl of 10 nM reporter probe labelled with HRP to each well and incubation for a further 30 minutes. After a final washing step, the presence of HRP was measured following addition of 50 μl of TMB substrate, and 50 μl 1 M H_2_SO_4_ 5 minutes later. The absorbance was read at 450 nm (SpectraMax 340PC384, bioNova Scientifics S.L.). To check the sensitivity of the assay, amplification was carried out with different starting concentrations of DNA (100 nM, 10 nM, 1 nM, 0.1 nM, 0.01 nM, 0 nM). The Limit of Detection (LOD) was calculated using GraphPad Prism Software and is defined as the blank (no target) + 3 × SD of the blank. Triplicate measurements were performed for each concentration.

### Preparation of reporter probe-AuNPs conjugation

Gold nanoparticles (AuNPs) with an approximate average diameter of 13 nm were prepared by citrate reduction of HAuCl_4_, as previously described^70^. Conjugation of reporter DNA to AuNPs was achieved via mixture of 100 μl of reporter DNA probe with 1 ml of AuNPs. The solution was left to incubate for 24 hours at 1000 rpm in a thermomixer and salt was introduced every 20 minutes until a concentration of 0.7 M was reached. Subsequently, the mixture was again left to incubate for 24 hours under the same conditions. Finally, the conjugate was centrifuged at 15000 rpm for 30 minutes and the pellet was re-suspended three times in deionized water in order to clean the conjugate and to remove any free DNA. The conjugate was then re-suspended in deionized water to the desired volume. Additional functionalities were tested in an effort to increase the yield of conjugation such as co-immobilization with 6-mercapto-1-hexanol (MCH) (ratio DNA:MCH 1:10, 1:100) and pre-treatment of thiolated DNA with reducing agents (TCEP and DTT)^60^,^61^.

The conjugates were evaluated and characterised by Agarose gel and spectrophotometer. The gel was performed using ultra low pure agarose (3%) in 1× Tris-Borate-EDTA buffer (TBE) and run for 30 minutes at 100 V. To stain the gel, GelRed (Biotium, Barcelona, Spain) was used. Spectrophotomer (Cary 100 Bio UV-visible spectrophotometer, Agilent) was used to scan from 800 nm to 200 nm.

### Preparation of lateral flow test strip

The test strip was made by manually cutting in strips of 4 mm width. The membrane used was FF170HP nitrocelloluse (Whatman, Germany) and the absorbent pad was glass cellulose (Whatman, UK). The test and the control lines were prepared by drawing a line with an Eppendorf tip containing 20 pmol streptavidin and 60 pmol of the respective biotinylated probe in PBS buffer, which was then incubated for 1 hour at room temperature. Subsequently, the membrane was allowed to dry at room temperature for 1 hour, followed by a blocking step with 1% w/v skimmed milk powder and 0.1% w/v empigen detergent for 15 minutes, under shaking conditions. The membrane was left to dry, again at room temperature for approximately 2 hours and then stored in the fridge until use. The test strips were assembled according to Fig. 4a.

### Lateral Flow Assay

Ten microliters of reporter probe conjugated with AuNPs were mixed with 1 μl RPA product, and 8 μl of buffer solution (10× SSC, 3.5% v/v Triton X-100, 0.25% v/v SDS, 12.5% formamide to obtain a final concentration of 4× SSC buffer, 1.4% v/v Triton X-100, 0.1% v/v SDS, 5% formamide). The mixture was incubated for 3 minutes at RT before being wicked on to the test strip. One of the tails of the RPA amplicon formed a complex with the AuNP-DNA reporter probe, and this complex flowed along the nitrocellulose membrane towards the test line, where it was captured by the immobilised DNA probe complementary to the other tail of RPA product. The excess of reporter probe conjugated with AuNPs, unbound by RPA product, flowed pass the test tine and was captured at the control line by another DNA probe to ensure the correct operation of the assay. An absorbent pad functioned as a wick to maintain the flow rate and direction, preventing any back flow or fluid.

In order to test the sensitivity of the assay, RPA was carried out with different concentrations of DNA (300 nM, 30 nM, 3 nM, 0.3 nM, 0.03 nM, 0.003 nM, 0 nM). A Smartphone camera was used to take an image of the strip, and analysed using a smartphone application based on Image J software termed IJ_mobile to calculate the intensity of the bands. These values were plotted in GraphPad Prism software in order to obtain the LOD of the assay. The data was normalized by subtracting the value obtained for the blank measurement. Triplicate measurements were performed for each concentration and LOD was calculated by the formula bottom value + 3× standard deviation of bottom value.

### Data Availability

Data supporting this publication is openly available under an ‘Open Data Commons Open Database License’. Additional metadata are available at: http://dx.doi.org/10.17634/122638-2. Please contact Newcastle Research Data Service at rdm@ncl.ac.uk for access instructions.

## Additional Information

**How to cite this article**: Jauset-Rubio, M. *et al*. Ultrasensitive, rapid and inexpensive detection of DNA using paper based lateral flow assay. *Sci. Rep.* **6**, 37732; doi: 10.1038/srep37732 (2016).

**Publisher's note:** Springer Nature remains neutral with regard to jurisdictional claims in published maps and institutional affiliations.

## Supplementary Material

Supplementary Information

## Figures and Tables

**Table 1:** Definition of ASSURED diagnostics.

Affordable – for those at risk of infection
Sensitive – minimal false negatives
Specific – minimal false positives
User-friendly – minimal steps to carry out test
Rapid & Robust - short turnaround time and no need for refrigerated storage
Equipment-free – no complex equipment
Delivered – to end users

**Table 2:** Comparison of Lateral Flow Assay Costs.

Strategy	Test line	Control line	Conjugate	Price
Tailed primers	Biotin-DNA	Biotin-DNA	AuNPs-Thiol-DNA	1, 15 €/strip (assay)
Labelled primers & antibodies	Anti-biotin antibody	Anti-rabbit IgG antibody	AuNPs-anti-FITC antibody	5, 22 €/strip (assay)
Labelled primers & antibodies	Streptavidin	Anti-rabbit IgG antibody	AuNPs-anti-FITC antibody	4, 14 €/strip (assay)
Milenia Hybridetect kit	Biotin-ligand	Polyclonal anti-rabbit antibody	AuNPs-anti-FITC antibody	3, 12 €/strip (assay) + 1, 25€ (FAM-probe + Biotin-primer)

**Table 3:** Sequences used in this study.

Name	Sequence
Capture probe maleimide plates	5′-gtcgtgactgggaaaacttttttttttttttt-C6 thiol-3′
Reporter probe maleimide plates	5′-HRP-actggccgtcgttttaca-3′
Capture probe test line	5′- gtcgtgactgggaaaacttttttttttttttt-Biotin-TEG-3′
Capture probe control line	5′-tgtaaaacgacggccagtttttttttttttttt-Biotin-TEG-3′
Reporter probe lateral flow (DNA 2)	5′-actggccgtcgttttacattttttttttttttt-C6 thiol-3′
Reporter probe lateral flow (DNA 1)	5′-actggccgtcgttttaca-C6 thiol-3′
Duplex DNA	5′agctccagaagataaattacaggggccggggtggctcaggcaaggggttgacctgt 3′tcgaggtcttctatttaatgtccccggccccaccgagtccgttccccaactggaca cgtagggattgttttaacaactaggatactatgacccc-3′ gcatccctaacaaaattgttgatcctatgatactgggg-5′
Forward primer	5′-gttttcccagtcacgac-C3-agctccagaagataaattacagg-3′
Reverse primer	5′-tgtaaaacgacggccagt-C3-ggggtcatagtatcctagttg-3′
