# Supplementary material for: Ultrasensitive, rapid and inexpensive detection of DNA using paper based lateral flow assay
Source: Sci Rep. 2016 Nov 25;6:37732. doi: 10.1038/srep37732 (PMC5123575; doi:10.1038/srep37732)
Supplement: Supplementary Information [file srep37732-s1.doc]

**SUPPLEMENTARY INFORMATION**

**Ultrasensitive, rapid and inexpensive detection of DNA using paper based lateral flow assay**

*Miriam Jauset-Rubio ^a^, Markéta Svobodová ^a^, Teresa Mairal ^a^, Calum McNeil ^b^, Neil Keegan ^b^*, Ayman Saeed ^c^, Mohammad Nooredeen Abbas ^c^, Mohammad S. El-Shahawi ^d^, Abdulaziz S. Bashammakh ^d^, Abdulrahman O. Alyoubi ^d^ and Ciara K. O´Sullivan ^a,e^**

^a^ Nanobiotechnology and Bioanalysis group, Department of Chemical Engineering, Universitat Rovira I Virgili, 43007 Tarragona, Spain

^b^ Institute of Cellular Medicine, Diagnostic and Therapeutic Technologies Group, Newcastle University, Newcastle upon Tyne, NE2 4HH, UK

^c^ National Research Centre, Cairo, Egypt

^d^ Department of Chemistry, Faculty of Science, King Abdulaziz University, P. O. Box 80203, Jeddah 21589, Kingdom of Saudi Arabia.

^e^ Institució Catalana de Recerca I Estudis Avancats, Passeig Lluís Companys 23, 08010 Barcelona, Spain

* To whom correspondence should be addressed. Tel: [0034977558740]; Fax: [0034977559667]; Email: [ciara.osullivan@urv.cat]. Correspondence may also be addressed to Tel. [[0044 (0) 191 208 3678](tel:%2B44 (0) 191 208 3678)]; Fax: [[0044 (0) 191 208 7991](tel:%2B44 (0) 191 208 7991)]; Email: [neil.keegan@newcastle.ac.uk].


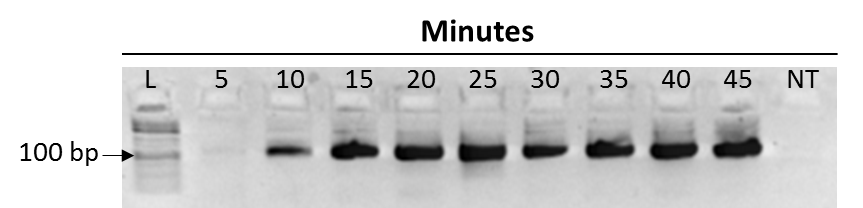


**Supplementary Figure S1.** Time-dependent RPA assay. L corresponds to ladder (10 bp DNA ladder Invitrogen); NT to non-template control.

**Supplementary Table S1.** Maleimide coated microtiter plate assay: Statistics values.

| **DNA (nM)** | **Replicate 1** | **Replicate 2** | **Replicate 3** | **Average** | **SD** |
| --- | --- | --- | --- | --- | --- |
| 100 | 2.6010 | 2.6300 | 2.5987 | 2.6099 | 0.0174 |
| 10 | 2.1100 | 2.0850 | 2.0503 | 2.0817 | 0.0299 |
| 1 | 1.6468 | 1.6601 | 1.5987 | 1.6352 | 0.0323 |
| 0.1 | 1.0245 | 0.9102 | 0.9975 | 0.9774 | 0.0597 |
| 0.01 | 0.5001 | 0.5601 | 0.5763 | 0.5455 | 0.0401 |
| 0.001 | 0.3134 | 0.3409 | 0.3331 | 0.3291 | 0.0141 |
| 0 | 0.0640 | 0.0590 | 0.0562 | 0.0598 | 0.0040 |

**Supplementary Table S2.** Lateral flow assay: Normalised statistics values.

| **DNA (nM)** | **Replicate 1** | **Replicate 2** | **Replicate 3** | **Average** | **SD** |
| --- | --- | --- | --- | --- | --- |
| 300 | 30087 | 29000 | 29500 | 29529.00 | 544.08 |
| 30 | 26609 | 26754 | 27000 | 26787.66 | 197.66 |
| 3 | 24132 | 24476 | 25000 | 24536.00 | 437.09 |
| 0.3 | 18196 | 17921 | 18700 | 18272.33 | 395.07 |
| 0.03 | 6007 | 8000 | 7500 | 7169.00 | 1036.91 |
| 0.003 | 1369 | 1260 | 1300 | 1309.66 | 55.14 |
